# Endocardial Endothelial Dysfunction Progressively Disrupts Initially Anti then Pro-Thrombotic Pathways in Heart Failure Mice

**DOI:** 10.1371/journal.pone.0142940

**Published:** 2015-11-13

**Authors:** Amanda Schoner, Christina Tyrrell, Melinda Wu, Jill M. Gelow, Alicia A. Hayes, Jonathan R. Lindner, Kent L. Thornburg, Wohaib Hasan

**Affiliations:** Knight Cardiovascular Institute, Oregon Health and Science University, Portland, Oregon, United States of America; Nagoya University, JAPAN

## Abstract

**Objective:**

An experimental model of endocardial thrombosis has not been developed and endocardial endothelial dysfunction in heart failure (HF) is understudied. We sought to determine whether disruption of the endothelial anti-coagulant activated protein C (APC) pathway in CREB_A133_ HF mice promotes endocardial thrombosis in the acute decompensated phase of the disease, and whether alterations in von Willebrand factor (vWF) secretion from HF endocardium reduces thrombus formation as HF stabilizes.

**Approach and results:**

Echocardiography was used to follow HF development and to detect endocardial thrombi in CREB_A133_ mice. Endocardial thrombi incidence was confirmed with immunohistochemistry and histology. In early and acute decompensated phases of HF, CREB_A133_ mice had the highest incidence of endocardial thrombi and these mice also had a shorter tail-bleeding index consistent with a pro-thrombotic milieu. Both APC generation, and expression of receptors that promote APC function (thrombomodulin, endothelial protein C receptor, protein S), were suppressed in the endocardium of acute decompensated HF mice. However, in stable compensated HF mice, an attenuation occurred for vWF protein content and secretion from endocardial endothelial cells, vWF-dependent platelet agglutination (by ristocetin), and thrombin generation on the endocardial surface.

**Conclusions:**

CREB_A133_ mice develop HF and endocardial endothelial dysfunction. Attenuation of the anti-coagulant APC pathway promotes endocardial thrombosis in early and acute decompensated phases of HF. However, in stable compensated HF mice, disruptions in endothelial vWF expression and extrusion may actually reduce the incidence of endocardial thrombosis.

## Introduction

Some 60% of heart failure (HF) patients suffer thromboembolic complications that are a leading cause of mortality and morbidity [1]. Thrombogenesis is likely promoted in HF due to endothelial damage, alterations in blood constituents, and low cardiac output [2, 3]. Anti-coagulant therapy carries bleeding risks and is only recommended for HF patients, presenting without prothrombotic atrial fibrillation, when clear thromboembolic risk factors are present such as presence of a left ventricular (LV) thrombus [4]. However, these thrombi are frequently quite small which makes them difficult to detect and increases their likelihood of embolization [5, 6]. Estimates for the prevalence of LV thrombi in heart failure patients range from 11–44% with echocardiography and magnetic resonance imaging (MRI) detection [7–12]. For patients with idiopathic dilated cardiomyopathy, the prevalence of LV thrombi may be as high as 69% [6, 11, 13–15]. Despite the serious thromboembolic risks in HF patients, a clear understanding of the etiology, structure and progression of endocardial thrombi is lacking. In this context, an animal model that reliably recapitulates stages of heart failure, and significant incidence of LV thrombi, is likely to be critical for understanding the pathogenesis of endocardial thrombosis.

We focus in this study on CREB_A133_ mice that have a dominant negative mutation (Ser-Ala^133^) in the cyclic AMP response element binding protein (CREB) within cardiomyocytes. As a result of this mutation, CREB-dependent transcriptional events within cardiomyocytes are severely disrupted, mitochondrial dysfunction occurs [16], and the resultant oxidative damage results in development of idiopathic dilated cardiomyopathy, endothelial dysfunction, and eventual HF [17, 18]. As HF develops in these mice, contractile responses to adrenergic stimulation are attenuated [17]. This model is therefore similar to clinical HF where adrenergic signaling via cyclic AMP is also disrupted and the resultant hyperadrenergic signaling is critical for HF development [19, 20]. The CREB_A133_ model is also unique in that direct chemical, infective or ischemic damage is not required for endocardial thrombi formation. Further, this model has significant advantages over surgical ligation models that have inherent variability in infarct size as well as the confounding effects of inflammatory cells and cytokines. We have used these CREB_A133_ mice to systematically investigate the link between progressive endocardial dysfunction and thrombus formation.

Endocardial endothelial cells (EECs) that line the cardiac chambers share many properties with vascular endothelium but they differ in that they have distinct developmental origins, have an abundance of gap junctions (sensory function), and are critical for cardiomyocyte peak force development through secretion of cardioactive factors (e.g. endothelin-1 and nitric oxide) [21, 22]. Although endocardial thrombi can develop in HF, an open question is whether EEC anticoagulant function is compromised in various stages of HF.

The endothelium is normally kept thrombus free through generation of activated protein C (APC) on its luminal surface [23, 24]. APC forms when circulating thrombin binds to the endothelial thrombomodulin receptor (TM) and activates PC. This reaction is facilitated by the endothelial PC receptor (EPCR) that binds PC then presents it to the TM-thrombin complex [23, 24]. APC, a serine protease, can inactivate the key coagulation factors Va and VIIIa in tandem with its cofactor Protein S. A delicate balance normally exists allowing coagulation to occur when necessary while keeping thrombosis at bay. Decreased PC expression, or mutations in TM, increases the risk for vascular and atrial endocardial thrombus formation [25–27]. Indeed, decreased APC activation in the left atrial endocardium may promote thrombosis in atrial fibrillation [26]. Therefore, regulation of endothelial TM and EPCR is a critical part of endothelial cell anticoagulant function that may be disrupted in HF.

Controlling APC generation is one of the key mechanisms used by endothelial cells to suppress the coagulation cascade; these cells can also promote coagulation through their synthesis and release of von Willebrand factor (vWF). When large multimeric vWF chains are released from intracellular Weibel-Palade bodies, platelets more easily adhere to the endothelial surface [28]. Dysfunctional endothelial cells are known to increase vWF secretion rates [29].

This study introduces an animal model in which early, acute decompensated, and stable compensated stages of HF can be investigated. Using the model we tested two hypotheses: 1) A prothrombotic endocardial surface characterizes the early and acute decompensated phases of HF as APC generation is attenuated and vWF synthesis and secretion are augmented. 2) The incidence of endocardial thrombus formation is reduced in stable compensated HF mice.

## Materials and Methods

### CREB_A133_ model of heart failure

The heart failure (HF) mice used in this study were transgenic CREB_A133_ mice that have a dominant negative mutation (Ser-Ala^133^) in the cyclic AMP response element binding protein (CREB) within cardiomyocytes (originally from Dr. Eric Svensson, University of Chicago). CREB-dependent transcriptional events within cardiomyocytes are severely disrupted in these mice resulting in idiopathic dilated cardiomyopathy and eventual HF [17, 18]. About 40% of CREB_A133_ mice developed acute decompensated symptoms of congestive HF between 10–20 weeks (*acute decompensated HF phenotype*) with visible thoracic and abdominal anasarca, labored breathing, decreased activity, and dilated ventricular chambers and nutmeg liver on necropsy. HF mice with the acute phenotype were euthanized 24–48 hr after onset of significant anasarca as otherwise they were prone to sudden cardiac death. From 10–14 weeks, a mild form of the disease existed (*early HF phenotype*). CREB_A133_ mice that did not develop the acute HF phenotype were sacrificed between 20–24 weeks (*chronic compensated HF phenotype*). CREB_A133_ mice were on a CD-1 background, and wildtype CD-1 breeder females were obtained from Charles River Laboratories (Wilmington, MA). Both male and female mice were used in this study. Since evidence exists that HF phenotype may be more extreme in female CREB_A133_ mice [16], we were particularly careful to keep an equal number of males and females in each group. Wildtype littermates to CREB_A133_ mice served as controls. No significant differences were seen between wildtypes from different ages and their data is presented as a combined pool. All mice were housed in a temperature controlled room (22±2°C) set to a 12-hr dark/light cycle and provided chow and water ad libitum. All experimental protocols were conducted in agreement with the NIH’s Guide for the Health and Use of Laboratory Animals, and approved by the Oregon Health & Science University Institutional Animal Care and Use Committee (protocol: IS00001994).

### Echocardiography

Transthoracic echocardiography (Vevo-2100, VisualSonics, Ontario, Canada) was performed in the parasternal long axis and mid-ventricular short axis planes using a high-frequency (40MHz) phased-array transducer (n = 7–9 per group). Mice were anesthetized under isoflurane and, in order to measure dimensions precisely at end-systole and end-diastole, two-dimensional acquisitions were performed at a high frame (approximately 1,000 Hz) by ECG gating of sequential M-mode sweeps. Diastolic dimension of the septal and posterior walls, and the LV internal diameter at end-diastole (LVID_d_) and end-systole (LVID_s_) at the minor axis were measured at the mid ventricular level. Left ventricular ejection fraction (LVEF) was calculated from cavity and wall dimensions as previously described [30]. Stroke volume (SV) was measured by the product of the LV outflow tract area and angle-corrected time-velocity integral measured by pulsed-wave Doppler from a high right parasternal window. Cardiac output (CO) was calculated as a product of heart rate and stroke volume, both of which were expressed relative to animal weight.

### Histology and immunohistochemistry

Hearts (n = 6 per group) were fixed in 4% paraformaldehyde, cryoprotected then cryosectioned in 3-stepped series of 8 slides per set (10 μm sections, 8 serial sections per slide) through the ventricles at the basal, medial and apical aspects giving a representative cross-section through the ventricles. One set of slides (n = 6 per group) were double immunostained with antibodies directed against vWF (rabbit anti-human, Santa Cruz) and fibrin β-chain (mouse anti-human, Sekisui, Stamford, CT). A second set was doublestained for CD31 (goat anti-mouse, Santa Cruz) and vWF/fibrin/urokinase plasminogen activator (rabbit anti-human, Santa Cruz). Images were visualized with a confocal microscope (Nikon A1R) with orthogonal projection of z-stack images carried out with NIS Elements software (Nikon Instruments, Melville, NY). Quantitation of vWF immunostaining was carried out with ImageJ (NIH); threshold discrimination for staining area expressed relative to total sectional area [31, 32].

For histology, hearts (n = 4 per group) were fixed in formalin then paraffin processed and sectioned (6 μm) followed by hematoxylin and eosin or Masson’s trichrome (Sigma-Aldrich) staining with standard histological protocols.

### Thrombin and APC generation assay

#### Thrombin generation assay

Mice (n = 4–7 per group) were anesthetized with a ketamine (75 mg/kg)/ acepromazine (2 mg/kg)/ xylazine (15 mg/kg) cocktail and the hearts immediately removed after thoracotomy. The atria were dissected free of the ventricles and the LV cut medially along its longitudinal axis to form two halves. Each LV half was placed over a well on a modified 384-well plate, endocardial surface exposed, and incubated with factors (HaemTech; Essex Junction, Vermont): prothrombin (50 nM), Va (50 nM), and Xa (5 nM) in HBS buffer (Hepes 25 mM; NaCl 140 mM; Na_2_HPO_4_ 0.75 mM; CaCl_2_ 5mM) for 20 min at 37°C (75 μl per well). After incubation, 50 μl was removed from each ventricle piece and placed in a 384-well read plate, and thrombin activity inhibited in one well with 5 μl Hirudin (200 ATU/mL, Sigma Aldrich, Saint Louis, MO) for 5 min at 37°C. Addition of 5 μl of 1 mM S-2238 chromogenic substrate (Diapharma, West Chester, OH) to each well was followed by photometer (VMax; Molecular Devices, Sunnyvale, CA) measurements of the rate of substrate cleavage at 405 nm by thrombin. A standard curve with known concentrations of α-thrombin (0.039–25 nM) was run at the same time as experimental samples. Thrombin generation was expressed as a delta change from wells where thrombin activity was inhibited by Hirudin versus wells where thrombin generation was not inhibited. Data for APC and thrombin generation assays was normalized to LV weight as both LV and heart weights were higher in acute and chronic stable HF mice (data not shown) compared to wildtype and early HF mice.

#### APC generation assay

Each half of the LV (dissected as above; n = 4–6 hearts per group) was placed over a well on a modified 384-well plate (as above; only endocardial surface exposed) and incubated with 75 μl of reaction buffer (Tris-HCl buffer, 50 mM; NaCl, 175 mM; CaCl_2_, 4 mM; BSA 0.1%) containing 10 nM human α-thrombin (HaemTech) and 1 μM human protein C (HaemTech) for 30 mins at 37°C. 50 μl was removed from each ventricle piece, placed in a 384-well read plate, and thrombin activity inhibited (as it can cleave the substrate) with 5 μl Hirudin (200 ATU/mL, Sigma Aldrich) for 5 min at 37°C. Addition of 5 μl of 2 mM S-2366 chromogenic substrate (Diapharma) to each well was followed by photometer measurements of the rate of substrate cleavage at 405 nm, which is proportional to APC activity. A standard curve with known concentrations of APC (25–3500 ng/ml, HaemTech) was run at the same time as experimental samples for concentration determinations. APC generation was expressed as a delta change from control wells containing all factors but not incubated with ventricular tissue. Data from technical repeat wells were averaged to obtain a mean value for APC generation from each animal’s left ventricle.

### Immunoblot and qRT-PCR analysis

#### qRT-PCR determination of TM, EPCR and Protein S

To extract RNA from the endocardial surface, LV halves (n = 5–7 per group) were placed over a modified 384-well plate (as above) and incubated with Trizol reagent (100 μL, 15 min, Life Technologies, Grand Island, NY) followed by a standard chloroform-ethanol extraction and purification (RNA Clean, Zymo Research, Irvine, CA) [31]. 15 min Trizol reagent incubation of the LV endocardial surface was chosen; this time frame gave the highest yield of RNA that was positive for CD31 as an endothelial marker and negative for the cardiomyocyte marker cardiac beta-myosin heavy chain. After RNA concentration determinations with a NanoDrop 2000 (Thermoscientific, Waltham, MA), cDNA was synthesized from 280 ng of total RNA (RT kit, Life Technologies) with a T100 thermal cycler (BioRad, Hercules, CA).

The cDNA was diluted 1:4 and a quantitative real-time PCR reaction carried out with Taqman Gene Expression Master Mix and mouse Taqman forward and reverse primers with a Viia7 qRT-PCR system (Life Technologies). Assays were carried out in triplicate (technical replicates) and with negative controls. Manufacturer certified Taqman probe/primer sets were to TM (Mm00437014), EPCR (Mm00440992), vWF (Mm00550376), protein S (Mm0134326), and to two housekeeping genes; CD31 (Mm01242584) and Polr2a (Mm00839493). Data are expressed as a delta change in Ct expression from that of the housekeeping gene Polr2a [31].

#### Immunoblot determination of TM, EPCR and Protein S

Protein was isolated from the endocardial surface of the LV (n = 4–9 per group) following incubation in Lysis-M buffer (with protease and phosphatase inhibitors; Roche Life Science, Indianapolis, IN) in a modified 384-well plate (37°C, 30 min) where only the endocardial surface was exposed to lysis buffer. 30 min lysis buffer incubation of the LV endocardial surface was chosen; this time frame gave the highest yield of protein that was positive for CD31 as an endothelial marker and negative for the cardiomyocyte marker cardiac beta-myosin heavy chain. A standard immunoblot protocol was followed as previously [31, 32] with 10 μg of protein loaded per well under reducing conditions with NuPAGE 4–12% Bis-Tris precast gels (Life Technologies). Following transfer to membrane, blots were incubated with antibodies to TM (goat anti-mouse, Santa Cruz), EPCR (rat anti-mouse, gift from Dr. Charles Esmon, Oklahoma Medical Research Foundation), protein S (rabbit anti-mouse, Santa Cruz), and housekeeping control Glyceraldehyde 3-phosphate dehydrogenase (GAPDH; rabbit anti-mouse, Sigma-Aldrich). Anti-HRP secondary antibodies were applied and protein bands visualized by chemiluminescence (FluorChem Imager, R&D) following incubation with SuperSignal West Dura substrate (ThermoScientific). Antibody specificity was determined by immunoblotting following loading of gels with naïve proteins (10–100 ng; TM from Santa Cruz, EPCR from LifeTech, protein S from HaemTech). Quantitation of band area was with ImageJ (NIH); band area is expressed as a percentage of that for GAPDH [31, 32].

Immunoblots for vWF were carried out as above except we used 3–8% tris-acetate gels (Life Technologies) under non-reducing conditions.

### Platelet aggregometry and tail clot assay

Following anesthesia (as above), blood was gently removed from the LV (n = 4–6 hearts per group) after cardiac puncture and centrifuged in a sodium citrate vial (150 rcf, 10 min) to obtain a platelet rich plasma fraction (PRP). A platelet poor plasma (PPP) fraction was obtained after a second centrifugation step (2,500 rcf, 20 min). PRP was diluted to 250, 000 platelets mm^3^ and an aggregometer (PAP 8E, BioData, Horsham, PA) used to measure ADP-induced platelet aggregation (50 μM, BioData, Horsham, PA), or agglutination measured after addition of Ristocetin (5 mg/mL, BioData) [33]; PPP sample as baseline light transmission (100%). The aggregation/agglutination per minute was measured as a delta change from the PPP sample. PRP treated with vehicle controlled for spontaneous aggregation.

For the tail clot assay (modified from [34]), anesthetized animals (as above) were weighed, then 10 mm of the tail was transected and the tail immersed in a 50 mL falcon tube containing saline at 37°C. The total bleeding time in a 20 min window was recorded. The animal was weighed (with transected tail tip) at the end of the experiment to calculate blood loss. Data are expressed as the bleeding index; the product of total bleeding time (sec) and blood loss (g) [34].

### vWF secretion assay

LV halves (obtained as above; n = 5–9 per group) were either stimulated with mouse thrombin (100 nM, HaemTech; 30 min in HBS buffer, 37°C) to promote maximal vWF secretion, or left unstimulated (vehicle control). Two technical replicates of 50 μl each were removed from each well and a sandwich ELISA carried out as per manufacturer's instructions (Abnova, vWF ELISA kit, Taipei, Taiwan) to quantitate vWF extruded from the LV endocardial surface. A standard curve with known vWF concentrations (2.5–80 mU) was run in parallel. Data from the technical replicate wells were averaged to obtain a mean value per mouse left ventricle. To measure endocardial vWF protein levels (n = 4 per group), the LV was placed endocardial surface up onto a modified 384-well plate and Lysis M buffer applied (75 mL, 30 min); lysate was centrifuged then 200 μg protein per sample was processed for vWF ELISA as above.

### Data analysis

Data was analyzed (Prism version 5.0, GraphPad, San Diego, CA) using one or two-way ANOVA (p<0.05) for multiple comparisons and a Newman-Keuls post-hoc test. All data were normally distributed and data graphically expressed as standard error of mean (+/- SEM).

## Results

### CREB_A133_ mice develop decompensated and compensated heart failure

**Fig 1:**
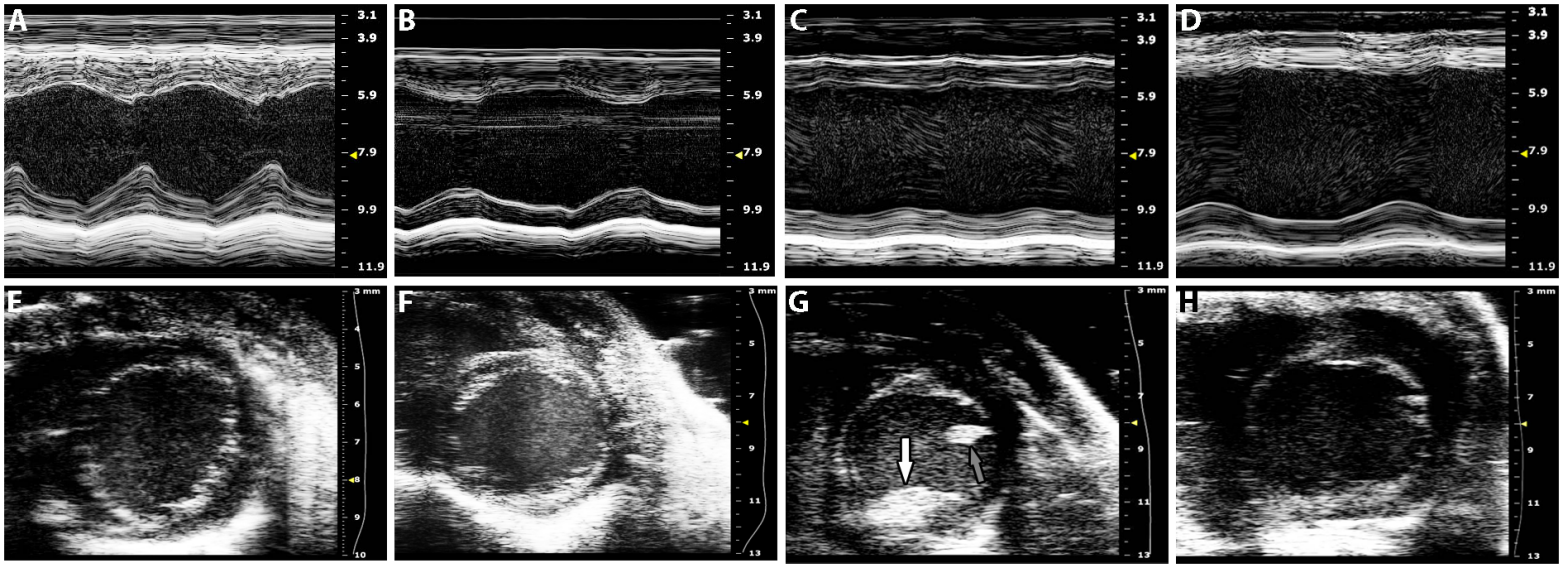
Representative M-mode (A-D) and short-axis views (E-H, at end diastole) following echocardiography of wildtype (A, E), early HF (B, F), acute HF (C, G), and chronic HF (D, H) mice. M-mode images were obtained from a parasternal long-axis view to assess septal (top) and posterior (bottom) wall thickening. A mural echolucent mass on the posterior wall consistent with mural thrombus is seen (G, white arrow) as well as papillary muscle (G, grey arrow). Speckle within LV cavities represents spontaneous echo contrast. Scale bar is on the right axis (mm).

**Fig 2:**
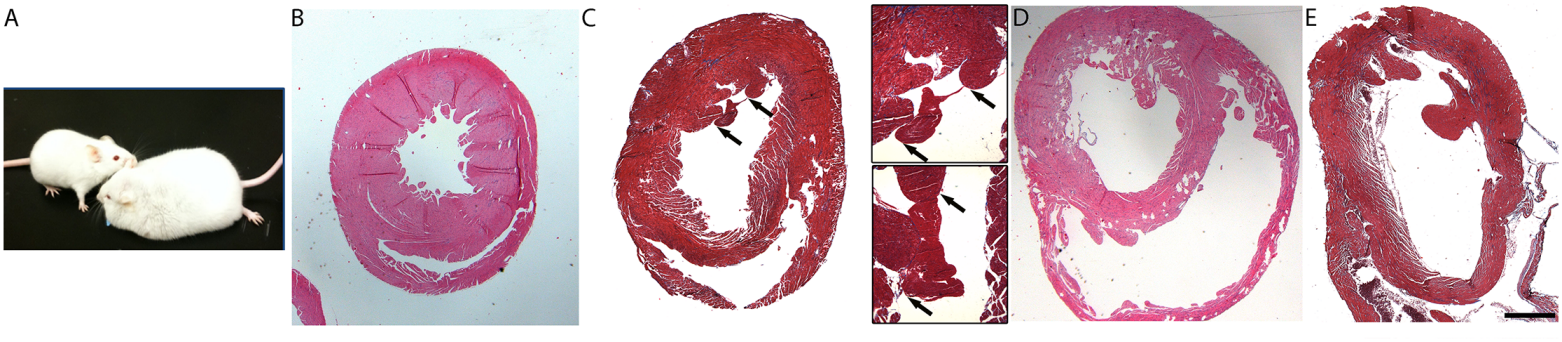
CREB_A133_ mice phenotype (A), and hematoxylin and eosin (B, D) and Masson’s trichrome (C, E) stained cross-sections through the ventricles of wildtype (B), early HF (C), acute HF (D), and chronic HF (E) mice. An acute HF mouse (A, right) is next to a wildtype littermate (A, left); note the significant anasarca in acute HF. In histological section, chambers are dilated and thinner with progression of HF. Insets in C show margins of an intracardiac thrombus in two different sectional planes. In the bottom inset panel, a fibrous band (blue) attaches the thrombus (150 μm x 100 μm) to the endocardium. Endocardial thrombi are present in early and acute HF mice with limited incidence in chronic HF. In E, post-surgical hemorrhage is seen within ventricles (orange/brown-staining with trichrome), without endocardial attachment. Scale bar in E is 1 mm for all panels (0.5 mm for insets).

We found three clearly demonstrable groups according to the timing and severity of their HF development; Early, Acute decompensated and Chronic compensated HF mice. Echocardiographic images of the CREB_A133_ mice (Fig 1) demonstrated LV dilation changes consistent with cardiomyopathy and LV systolic dysfunction. Although HF was present in all CREB_A133_ mice between 10–24 weeks, the most severe phenotype was in mice with sudden onset of significant anasarca between 10–20 weeks (*acute decompensated HF*; Fig 2A). These mice had, compared to wildtype mice, reductions in ejection fraction (LVEF; 52.4% reduction), cardiac output (CO; 54.4% reduction), fractional shortening (FS; 55.8% reduction) and fractional area change (FAC; 59% reduction, Table 1). Left ventricular internal diameters (LVID) were also increased in these acute HF mice at both diastole (LVIDd; 31.7% increase) and systole (LVIDs; 64.3% increase) compared to wildtype mice. CREB_A133_ mice at 10–14 weeks (*early HF*) and 20–24 weeks (*chronic compensated HF*) did not show overt symptoms of HF such as anasarca. However, both groups of mice did have underlying HF with dilated cardiomyopathy as evidenced by smaller LVEF and LVIDs compared to wildtype mice (Table 1). The degree of HF in both early and chronic HF mice was not as extensive as in acute HF mice, as LVEF was higher and LVIDs lower.

**Table 1:** Hemodynamic measurements from wildtype (WT) and CREB_A133_ mice during cardiac echocardiography.

	FAC (%)	FS (%)	LVEF (%)	SV (mL/mg)	CO (mL/min/mg)	LVIDd(mm)	LVIDs (mm)
Wildtype (n = 7)	50.2 ± 2.96	32.6 ± 1.56	63.4 ± 1.36	2.03 ± 0.26	952 ± 123	4.1 ± 0.21	2.8 ± 0.20
Early HF CREB_A133_ (n = 8)	32.54 ± 3.46 ***	23.9 ± 2.09 ###	47.7 ± 3.61 ***/###	1.65 ± 0.20	722 ± 81	4.4 ± 0.07 ###	3.4 ± 0.12 ***/###
Acute HF CREB_A133_ (n = 8)	20.6 ± 3.01 ***	14.4 ± 1.181 ***	30.2 ± 2.24 ***	1.55 ± 0.37	434 ± 57 **	5.4 ± 0.14 ***	4.6 ± 0.1 ***
Chronic HF CREB_A133_ (n = 9)	36.6 ± 5.03 ###	24.2 ± 2.82 ###	47.1 ± 4.73 ***/###	1.4 ± 0.18	590 ± 58 **	4.9 ± 0.19 ***	3.9 ± 0.13 *** /###

Fractional area change (FAC); fractional shortening (FS); left ventricular ejection fraction (LVEF); stroke volume (SV); cardiac output (CO); LV diastolic (d) and systolic (s) internal diameter (LVID).

*p<0.05 vs WT,

**p<0.01 vs WT,

***p<0.001 vs WT,

^#^p<0.05 vs Acute HF,

^##^p<0.01 vs Acute HF,

^###^p<0.001 vs Acute HF.

### Endocardial thrombi are prominent in ventricular chambers of early and acute decompensated HF mice

**Fig 3:**
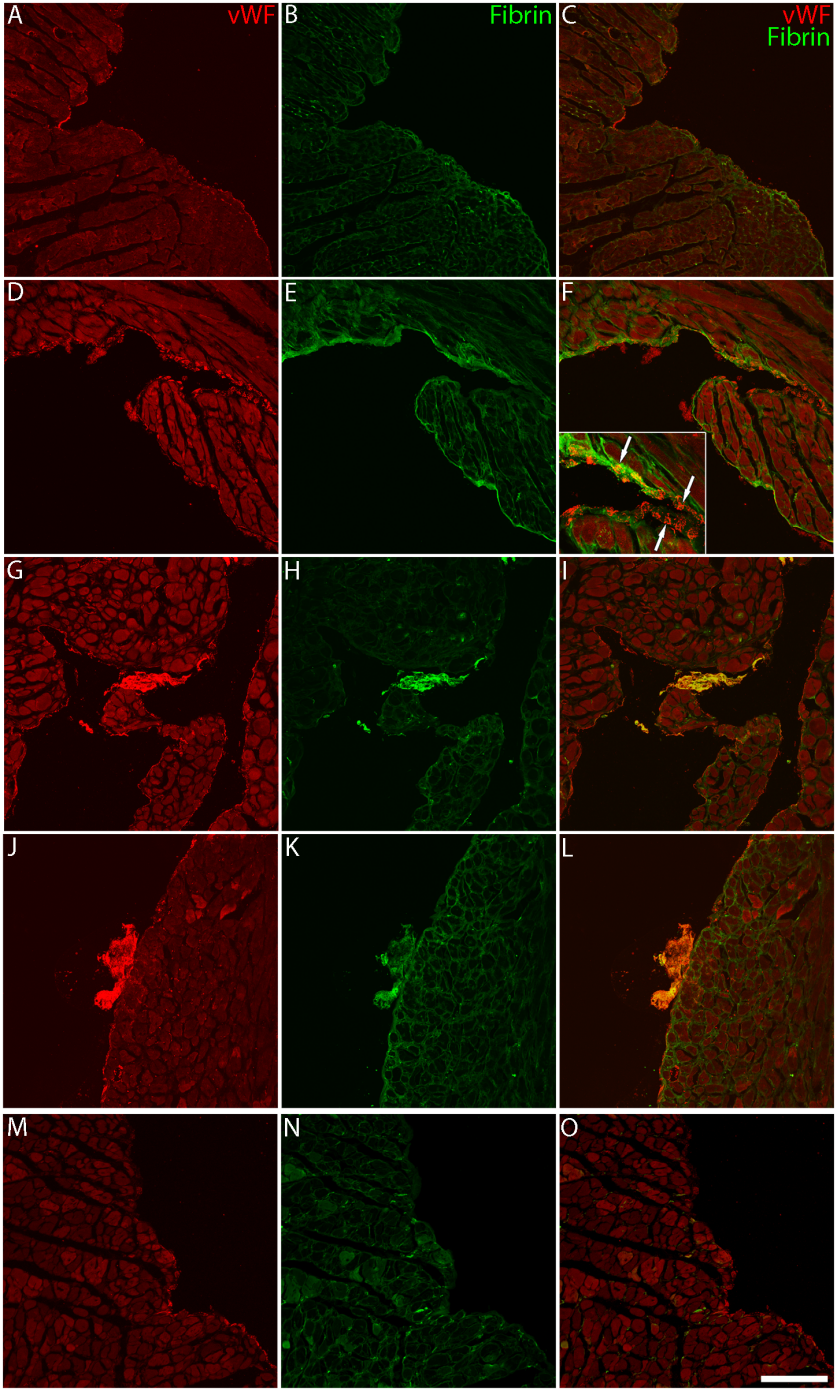
vWF (red; A,D,G,J,M), fibrin (green; B,E,H,K,N), and co-localized (C,F,I,L,O) photomicrographs of the LV. Images are from wildtype (A-C), early HF (D-I), acute HF (J-L), and chronic HF (M-O) mice. vWF staining is present within endocardial cells (within presumptive Weibel-Palade bodies) in all groups and very prominently in early HF (D, F, inset, arrows). Fibrin staining is limited on the endocardial surface of wildtype mice (B) but increased in HF mice (E, H, K, N). Small thrombi positive for both fibrin and vWF are present on the endocardial surface in both acute and chronic HF. vWF staining is limited on the endocardial surface in chronic HF mice (M-O). Note the heterogeneity in muscle fiber size in HF mice (I, O). Scale bar in O is 145 μm for all panels, except inset (84 μm).

While endocardial thrombi were sometimes detected by echocardiography (Fig 1E–1H), appearance in histological sections was more consistent. In sections, endocardial thrombi were seen in both early and acute decompensated HF mice but not in wildtype mice, and rarely in chronic compensated HF mice. LV thrombi incidence (present or absent) was as follows: 6/9 in early HF, 4/10 in acute HF, 1/8 in chronic HF. Large thrombi (1,100 μm x 650 μm, Fig 2C), attached to the endocardial surface, were observed, but rarely. Most intracardiac thrombi discovered in histological sections were smaller than the larger ones mentioned above but had dimensions greater than the minimum defined as 150 μm x 100 μm (Fig 3I and 3L); these were likely indistinguishable from trabeculae with conventional echocardiography. Mural thrombi were attached to the endocardial surface for most of their length, although some were pedunculated extending into the cavity. Endocardial thrombi were positive for both vWF and fibrin (Fig 3G–3L), and endothelial areas around thrombi were positive for urokinase plasminogen activator (data not shown).

Immunostaining for vWF (Fig 3) demonstrated localized expression of vWF on the endocardial surface and lining blood vessels in the myocardium of wildtype tissue. This expression pattern was maintained in early and acute HF mice, but was sparse in chronic HF mice. In early (Fig 3, panel D) and acute HF, endocardial endothelial cells had marked upregulation of vWF. Restriction of vWF staining to endothelial cells (both vascular and endocardial) was confirmed by double-staining with the endothelial markers CD31 and VE-cadherin (data not shown). Fibrin staining (Fig 3) was weak on the endothelial surface in wildtype mice and expression was greatly enhanced in HF mice.

### Thrombin generation is increased and APC generation attenuated on the endocardial surface of HF mice

**Fig 4:**
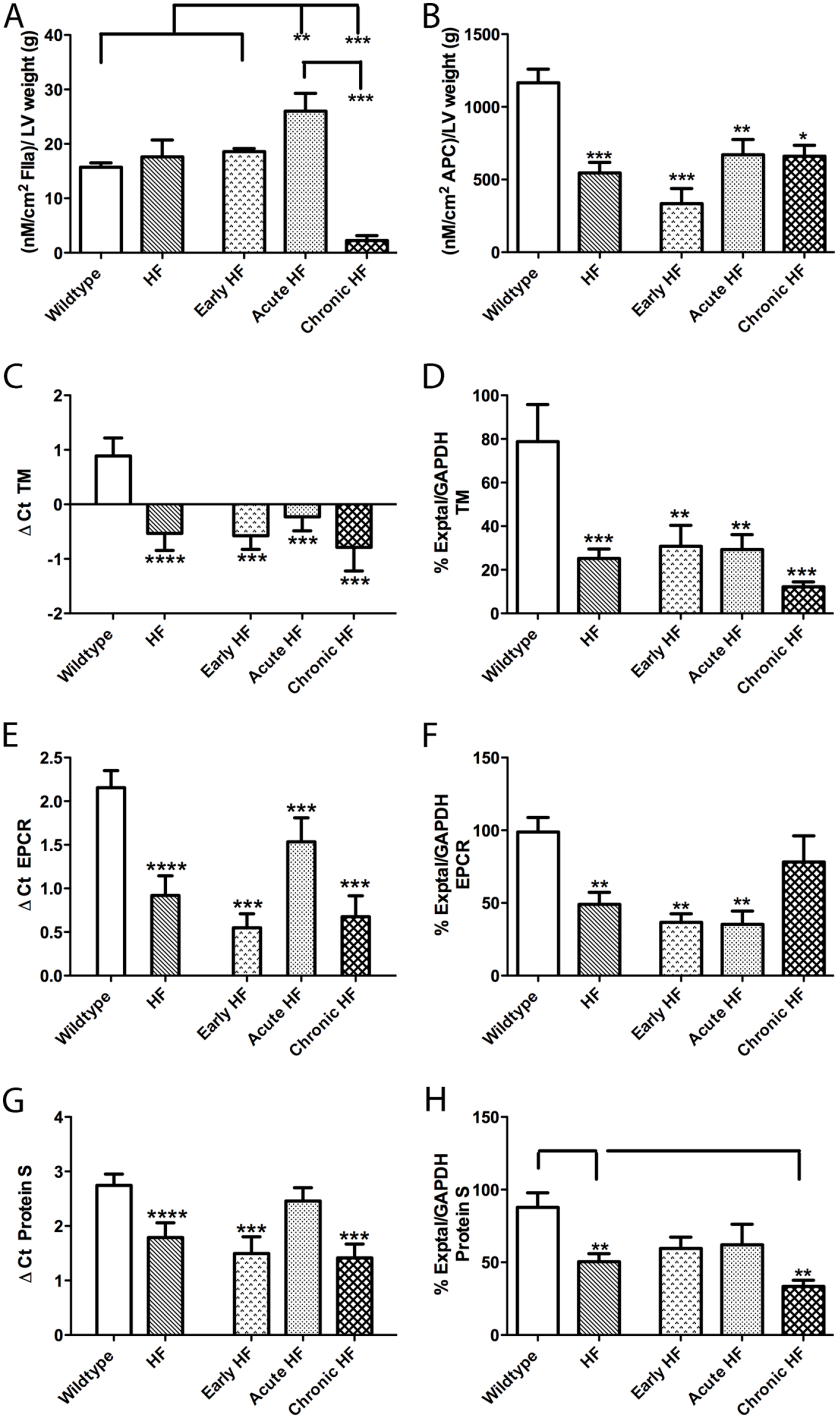
Thrombin (FIIa; A) and APC (B) generation, and TM (C, D), EPCR (E, F), and Protein S (G, H) expression, on the endocardial surface of wildtype, all HF (HF), early HF, acute HF, and chronic HF mice. Thrombin generation is increased in acute HF compared to wildtype mice (A). APC generation is decreased in all HF mice compared to wildtype mice (B). TM (C) and EPCR (E) mRNA expression is attenuated in all HF mice groups compared to wildtype mice. Protein S (G) transcripts are also decreased in early and chronic HF mice. Protein expression of TM (D) and Protein S (H) is decreased in chronic HF compared to wildtype mice. EPCR (F) protein expression is attenuated in early and acute HF mice relative to wildtype and chronic HF mice. *p<0.05, **p<0.01, ***p<0.005.

Activity assays for thrombin generation on the endocardial surface showed an increase in thrombin (FIIa) generation in acute HF compared to both wildtype (65.5%) and early HF (40%) mice (Fig 4A). In contrast, chronic HF mice had lower FIIa generation than all other groups (85–91% decreases). APC generation on the endocardial surface was attenuated in all HF groups (by 71.4% in early, 42.5% in acute, 43.3% in chronic HF) compared to wildtype LVs (Fig 4B).

### Thrombomodulin, EPCR and protein S expression on the endocardial surface is decreased in HF mice

Endocardial thrombomodulin (TM) mRNA and protein expression was decreased substantially (125–189% for mRNA, 61–84% for protein) in all HF mice groups compared to wildtype mice (Fig 4C and 4D). A similar profile of lowered mRNA (29–74.5%) and protein (21–64%) was seen for endocardial EPCR expression (Fig 4E and 4F), although the chronic HF group was not statistically different from wildtypes for EPCR protein. A general trend for attenuated mRNA and protein in HF mice was also seen for protein S expression, with the chronic HF group substantially decreased (48% for mRNA, 62% for protein) from wildtype mice (Fig 4G and 4H).

### Tail blood coagulation is quicker, but vWF-induced agglutination attenuated, in acute decompensated HF mice

**Fig 5:**
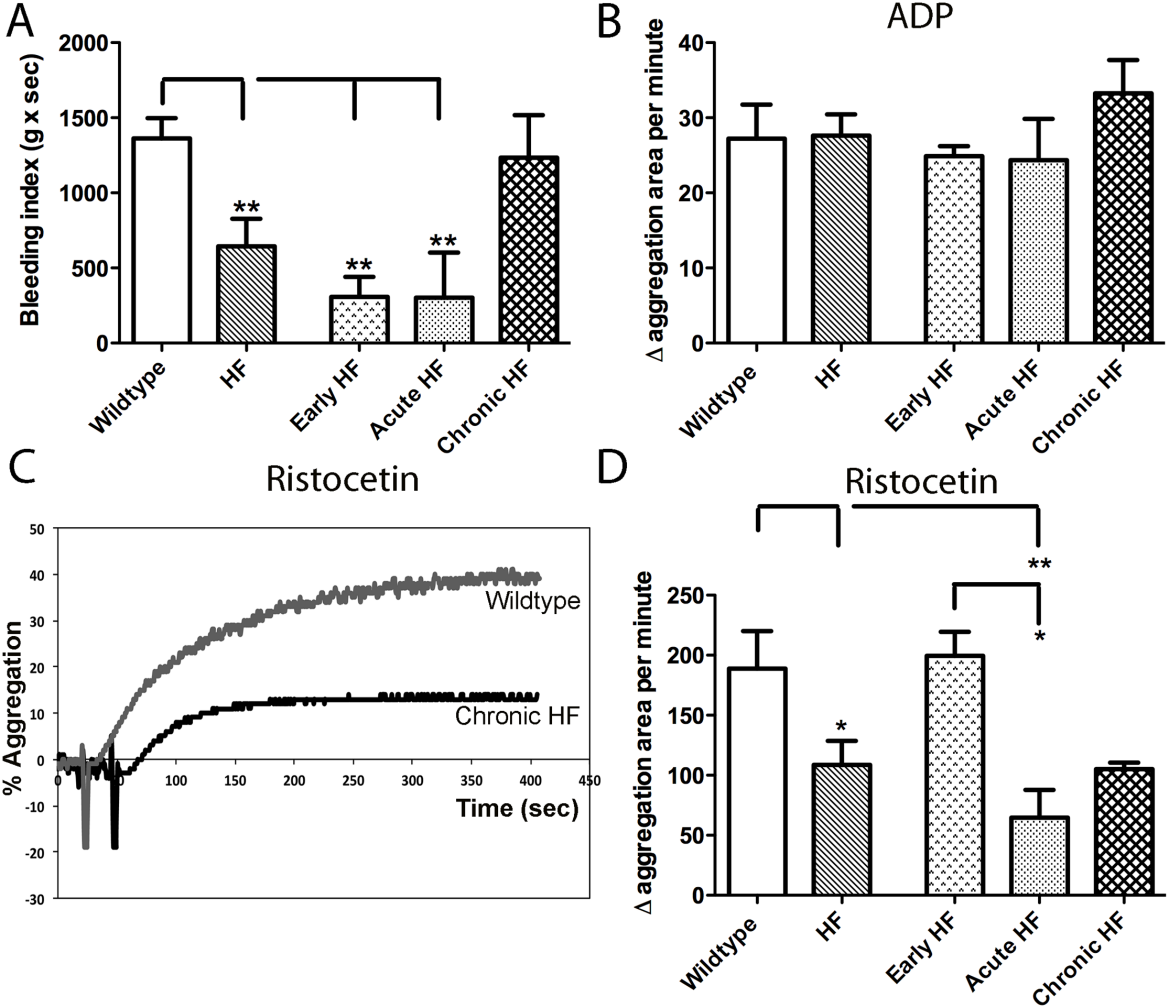
Tail clot assay (A) and platelet aggregometry (B-D) in wildtype, all HF (HF), early HF, acute HF, and chronic HF mice. The bleeding index, reflecting time to clot and total blood loss, is decreased in early and acute HF mice compared to wildtype and chronic HF mice (A; p<0.005). Platelet aggregation with ADP is similar across all groups (B). In contrast, platelet agglutination with ristocetin is attenuated overall in HF mice (p<0.05; representative trace in C, data in D), particularly in acute HF mice.

The bleeding index, reflecting time to clot and total blood loss, showed a significant decrease in clotting time in early and acute HF mice (77%) compared to wildtype mice (Fig 5A), consistent with a pro-thrombotic phenotype in these mice. Platelet aggregation with adenosine-5'-diphosphate (ADP) was similar between all groups of mice (Fig 5B). However, vWF-dependent platelet agglutination with ristocetin (Fig 5C and 5D) was lower in both acute (70%) and chronic (49%) HF mice.

### Circulating and endocardial endothelial vWF levels are elevated, but thrombin-induced vWF secretion is decreased, in acute decompensated HF mice

**Fig 6:**
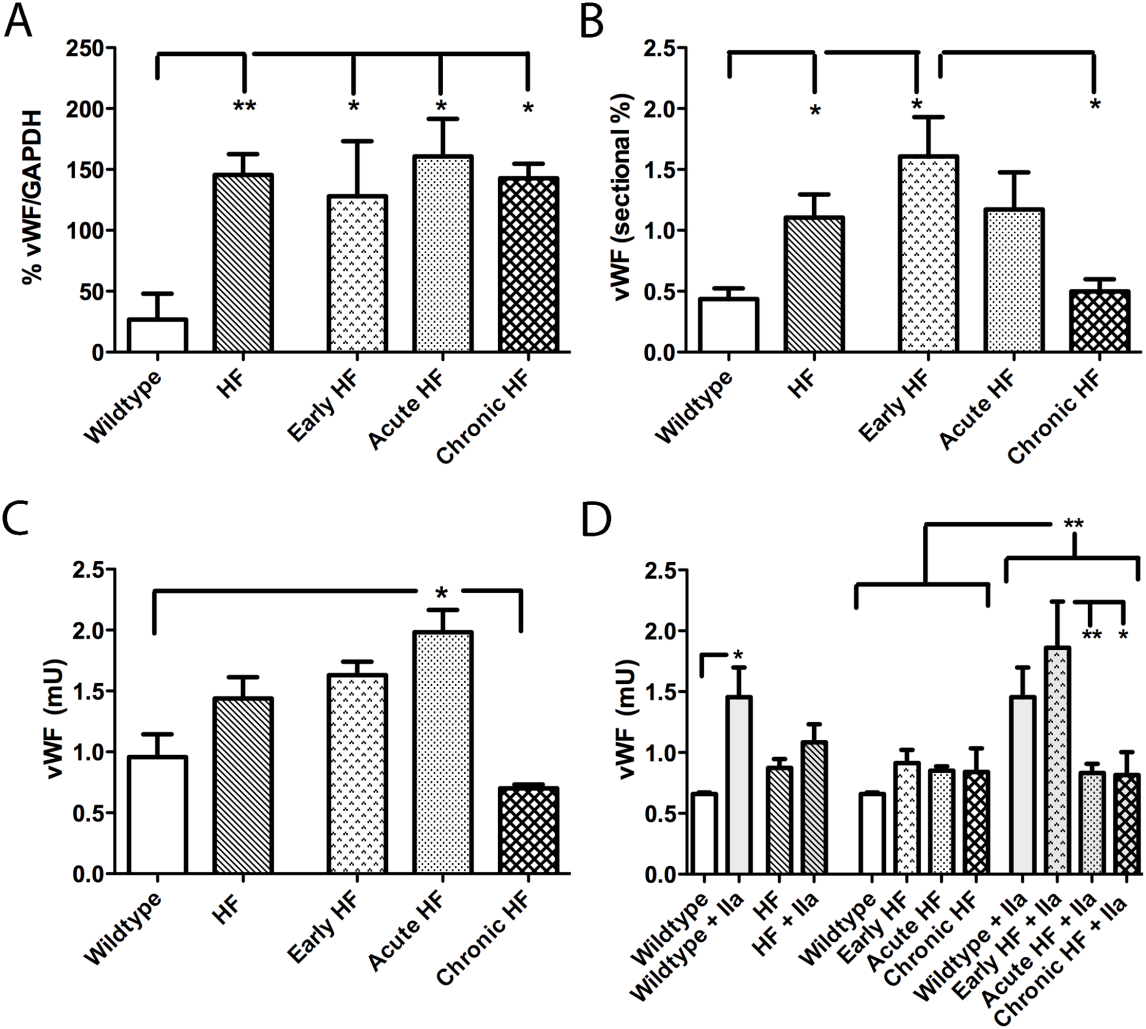
Quantitative determination of vWF: in plasma (A; immunoblot), by immunostaining in sectional endocardium (B; IHC), on the endocardial surface (C; ELISA), and following thrombin (IIa)-promoted vWF secretion from endocardium (D; ELISA). Circulating vWF expression is higher in all groups of HF compared to wildtype mice (A). Sectional percentage of vWF staining is higher in early HF compared to both wildtype and chronic HF mice (B). Endocardial vWF protein levels are higher in acute HF compared to both wildtype and chronic HF mice (C). Thrombin-promoted vWF extrusion is higher overall than unstimulated endocardium (D). Thrombin-promotion of vWF extrusion is prominent in wildtype mice and in early HF mice (D). *p<0.05, **p<0.01.

Plasma vWF showed a prominent 225 kDa vWF band that corresponded to the mature vWF protein. Mature vWF protein in plasma was increased in all HF mice groups by 378–500% over wildtype mice consistent with a pro-thrombotic phenotype (Fig 6A). Quantitation of vWF endocardial sectional staining showed a 267% increase in early HF mice over wildtype mice and, intriguingly, a 222% augmentation over chronic HF mice (Fig 6B). vWF protein extracted from the endocardial surface, reflecting both EEC and endocardial surface protein expression, was increased in acute HF mice compared to wildtype (107%; Fig 6C) and chronic HF (183%) mice. Addition of thrombin to the endocardial surface promoted vWF secretion in both wildtype (120% increase over baseline) and early HF (104% increase over baseline) mice (Fig 6D). However, for both acute and chronic HF mice, vWF extrusion levels did not change from pre-stimulation levels and were therefore lower (55%) than early HF post-stimulation levels.

## Discussion

We set out to establish a robust model of ventricular endocardial thrombosis using the CREB_A133_ transgenic mouse. Although models of atrial endocardial thrombosis exist for studies of atrial fibrillation [35, 36], to the best of our knowledge no model of HF is available that consistently demonstrates ventricular endocardial thrombi. Chemical or infective damage to the ventricles can promote endocardial thrombus formation [37]. However, direct damage to the endocardium does not recapitulate clinical HF where endocardial damage generally occurs secondary to cardiomyocyte failure or as a consequence of disturbed blood flow through the chambers. Similarly, with models of myocardial infarction [38], the role of inflammatory cells may be a critical variable that is hard to standardize given variability in infarct size with surgical approaches. In transgenic CREB_A133_ mice, cardiomyocyte disruption is the primary initiator of HF. We have shown increased incidence of organized LV thrombi in early and acute decompensated stages of HF in these mice. Although the size of ventricular thrombi was variable, this finding is similar to clinical investigations that have also observed a broad range in LV thrombus dimensions [6, 9]. A caveat with any quantitation of thrombus incidence is the propensity of these structures to embolize to other parts of the body. Nevertheless, our findings clearly demonstrate that thrombi were present within LV chambers, were numerous in early and acute decompensated HF, and were rich in both fibrin and vWF. In our model of HF therefore, development of dilated cardiomyopathy is accompanied by increased thrombus formation in the LV.

Endothelial dysfunction has been implicated in the increased risk for thrombus formation within the failing heart. Given the critical endogenous anticoagulant role of APC [23, 26], we examined APC generation on the LV endocardial surface. Our results show a striking decrease in the APC generating ability of the endocardium. This attenuation is paralleled by increased thrombin generation on the endocardial surface in acute decompensated HF mice. An attenuation of APC generation in the left atrial appendage, as compared to the right, has been postulated as a possible reason for the increased formation of thrombi within the left atria in atrial fibrillation [26]. In the latter study, however, thrombi numbers were not quantified. Our data are the first to link thrombus incidence with a depressed capacity of the endocardium to generate APC.

Having established reduced APC formation, and conversely increased thrombin generation, on HF mice endocardium, we asked whether reduced expression of the critical receptors involved in genesis of APC also occurred. Indeed, both TM and EPCR mRNA transcripts and protein were attenuated within the endocardium in both early and acute decompensated HF mice. Lower TM and EPCR expression has been shown in a number of pathological states to correlate with decreased APC generation and increased risk for thrombosis [39, 40]. The failing hearts of humans have increased secretion of the soluble form of the TM receptor which serves as a biomarker for HF progression [3, 41]. Increased soluble TM levels in the circulation occur in parallel with reduced endothelial TM expression, suggesting shedding of this receptor with endothelial dysfunction and coincident hypercoagulation [42]. In addition to TM and EPCR, we also examined endocardial expression of the APC co-factor receptor, protein S. Like the protein C receptors, protein S transcripts and protein expression were generally attenuated in HF mice. Overall, our data are consistent with endothelial dysfunction- associated decreases in APC generation in the failing mouse heart that are TM and EPCR-dependent.

Our APC pathway results are congruous with endothelial dysfunction in early and acute decompensated HF mice promoting a hypercoagulable endocardial phenotype. However, in chronic compensated HF mice, endocardial thrombin generation was markedly attenuated and they did not demonstrate an increased incidence of endocardial thrombi. Despite these indices of a hypocoagulable state, the APC pathway was severely disrupted in these chronic HF mice. We postulated that another endothelial-derived factor was responsible for an overall hypocoagulable phenotype in chronic HF mice. To this end, we examined vWF expression in, and thrombin-mediated vWF secretion from, the endocardial surface. As expected, vWF expression was increased in acute decompensated HF mice. However, thrombin-mediated vWF extrusion was markedly depressed in both acute decompensated and chronic compensated HF endocardium. At a functional level, such an attenuation of vWF extrusion would likely interfere with platelet aggregation and hence blood coagulation. Indeed, platelet agglutination with ristocetin, a vWF-dependent process, was also attenuated in both acute and chronic HF mice. That platelet function was normal in these mice was confirmed with normal aggregation responses to ADP. Finally, we examined tail bleeding to look at systemic effects on coagulation. Although early and acute HF mice showed hypercoagulation compared to wildtype mice, chronic HF mice actually had bleeding times similar to wildtype mice. We suspect that endothelial dysfunction in HF initially manifests itself as a prothrombotic state with defects in the APC pathway as a primary contributor. As HF progresses, however, the once increased vWF extrusion in early HF is gradually attenuated in chronic compensated HF mice, which may actually reduce the risk of endocardial thrombi in the chronic stable condition compared to the early phase.

It is not known whether there is a direct clinical relevance of HF stage-specific hypercoagulability followed by attenuated vWF secretion as shown in this mouse model. However, these studies suggest that a further understanding of the regulation of vWF, as a consequence of endothelial dysfunction, is needed in the clinical HF setting. Of interest in this context was the demonstration by Fukuchi et al [43] of a correlation between increased atrial vWF immunoreactivity and presence of atrial endocardial thrombi; intriguingly, this relationship was independent of concomitant atrial fibrillation. It is possible therefore that a similar relationship exists for HF patients, and that attenuated endothelial vWF secretion may in contrast correlate with reduced propensity for developing endocardial thrombi. Since many chronic heart failure patients are routinely prescribed either low dose aspirin or anticoagulants, whether these regimens may be masking progressive reductions in vWF secretion (and hence thrombus formation propensity) is an unknown that may require future investigations. Also of potential relevance here is the increased incidence of acquired von Willebrand deficiency in HF patients fitted with a LV assist device (LVAD) [44, 45]. Potential causes of this vWF deficiency may lie in the relative shear forces generated by axial vs. centrifugal LVAD devices [45]. However, the susceptibility of an already damaged endothelium to increased shear forces from LVAD devices could additionally result in decreased vWF secretion and thereby development of acquired von Willebrand syndrome.

Although chronic compensated HF mice displayed the strongest evidence for attenuated vWF secretion, the degree of attenuation likely depends on pathophysiologic factors that change as HF develops. While increased circulating vWF levels are regarded as adverse event predictors in the development of HF [46, 47], in stable compensated HF patients, plasma vWF levels are interestingly not elevated [48]. In acute decompensated HF mice, we saw overall a hypercoagulable phenotype. However, acute HF mice also showed, similar to chronic HF mice, attenuation in ristocetin-induced platelet agglutination and attenuated thrombin-induced vWF extrusion responses. Since acute HF mice had augmented circulating vWF levels, and an increased incidence of endocardial thrombi and tail bleeding index, it is likely that overall these mice were still in a hypercoagulable state despite these anomalous findings.

There are some limitations to our study. Since we were harvesting tissue from animals we were unable to examine the same animal for thrombus incidence at the early stage of HF versus acute or chronic stages of HF. Hence, we cannot state with certainty that animals in the chronic stages, that showed low incidence of endocardial thrombi, may have had endocardial thrombi in the early HF stage. Secondly, this study focuses on endothelial dysfunction in HF and its sequelae without examining the primary drivers of this endothelial dysfunction. There may be several mechanisms for endothelial dysfunction in these HF mice; these may include as a secondary consequence of cardiomyocyte death, or due to rheological changes within the failing cardiac chambers. However, these were not the primary research questions addressed by this study.

In conclusion, we were able to establish a model of endocardial dysfunction with consequent endocardial thrombosis. We found considerable evidence in favor of our first hypothesis that a prothrombotic endocardial surface characterizes the early and acute decompensated phases of HF, as APC generation is attenuated and vWF synthesis and secretion are augmented. We also generated data supporting our second hypothesis that in stable compensated HF, incidence of endocardial thrombus formation is reduced. However, the mechanism for this thrombus incidence reduction appears to be due to progressive endothelial disruption in chronic stages of the disease; this may correlate to chronic compensated HF patients that are thrombus free despite prolonged HF.

## Supporting Information

S1 Data Echo data. (XLSX)

S2 Data Thrombin activity assay data. (XLSX)

S3 Data APC activity assay data. (XLSX)

S4 Data qRT PCR data. (XLS)

S5 Data qRT PCR data. (XLS)

S6 Data qRT PCR data. (XLS)

S7 Data Immunoblot data. (XLSX)

S8 Data Tail clot assay data. (XLSX)

S9 Data Aggregometry data. (XLSX)

S10 Data vWF Immunoblot data. (XLSX)

S11 Data vWF sectional percent data. (XLSX)

S12 Data vWF ELISA endothelial surface data. (XLSX)

S13 Data vWF extrusion assay data. (XLSX)
